# An Examination of a Distorted Knee

**Published:** 1851-09

**Authors:** James Robertson

**Affiliations:** M. D., F.R.C.S., M.B., Lond., Physician to the Infirmary, Hitchin


### SURGERY.

*An examination of a distorted knee.* By JAMES ROBERTSON, M. D., F.R.C.S., M.B., Lond., Physician to the Infirmary, Hitchin.—I am induced to record the examination of the distorted knee of a person who lately died under my care (from ulceration of the intestines) because I find no such examination recorded; and from the growing importance of the surgical treatment of deformities, every item of pathological knowledge is desirable, and because it presented some interesting appearances, the knowledge of which may be useful when writing "On Deformities." Mr. Tamplin (and I know of no more experienced writer on the subject) says, (Lectures, pp. 120, 121, on Deformities of the Knee-joint,) "When the deformity is considerable, and has been so for some years, does the articulating surface itself alter in position; or does the internal condyle itself alter or increase in size, or project more than it does in its natural size? I do not believe that either of the changes takes place. I believe no alteration takes place; certainly none by attrition. Yet it is a question on which, from our present experience, I am unable to speak positively."

That these very alterations do *sometimes* take place, the following case proves.

R. P., aged 32, had his right knee distorted for many years; he attributed it to carrying water when young; it presented the usual appearance of genu valgum or knock-knee, of severe grade, the leg and thigh forming a considerable angle, which admitted of being slightly lessened by manipulation; his left leg was straight. After death the angle did not admit of alteration. The tendon of the biceps and the external portion of the fascia lata were tense, but division of these did not allow of any material alteration of the position of the leg. The internal lateral ligament was lengthened. On opening the joint, the interior, except the articular surface and the crucial ligaments, presented a dark purple color, from a layer of enlarged tortuous vessels gorged with blood, ramifying under the synovial membrane, which was disposed in an infinite number of folds hanging into the joint, in form after the fashion of the valvulæ conniventes, in consistence like loaded plexus choroides, showing, when magnified, innumerable compound—or looped—loops of vessels enveloped by synovial membrane. The joint contained from half to three quarters of an ounce of thick, glutinous, yellow transparent fluid. The articulating surfaces and crucial ligaments were of their natural color; a patch on the middle of the patella, as large as a sixpence, was partially denuded of cartilage, and had a puckered appearance. There was a similar but smaller spot on the edge of the articulating surface of each of the condyles in front; these places suggested the idea of old puckered cicatrices. The end of the external condyle, and corresponding portion of the articulating surface of the external tuberosity of the tibia were denuded of cartilage, and the cancellated substance of the bone was bared to an extent equal in size to a fourpenny piece; around these rough surfaces, for about the sixth of an inch, the cartilage was was detached and lay in a thin fringe on the bones. The cartilage became thicker as it receded from these fringes, and on the extremity of the inner condyle was scarcely less than a quarter of an inch thick.

On microscopic examination, these fringes consisted of little else than rows of cartilage cells. Whilst as the cartilage became thicker, the intercellular substance increased and the cells diminished in quantity, till at the thickest part these were thinly scattered. There was no appearance of vessels in the cartilage. The articulating surface of the inner condyle was considerably longer than natural, and more pointed; the outer, shorter and flatter. The semi-lunar cartilages were mere rings round the outside of the articular surface of the tibia; the crucial ligaments were perhaps rather long. The other parts of the joint presented nothing unusual. Thus, in this case, the internal condyle did project, was increased in size, the position of the articulating surfaces altered, and marked changes by attrition were evident, etc., etc., though not expected in such a case by the experienced writer above quoted. I need scarcely add, that joints similar to this would not promise a very successful result to operative or other efforts, to cure the deformity, and that I record this, not as offering the general condition of the parts in the same disease, but as showing what is sometimes their unpromising condition.—*Medical Times.*

